# Tumor-Derived Extracellular Vesicles Induce Abnormal Angiogenesis *via* TRPV4 Downregulation and Subsequent Activation of YAP and VEGFR2

**DOI:** 10.3389/fbioe.2021.790489

**Published:** 2021-12-23

**Authors:** Brianna Guarino, Venkatesh Katari, Ravi Adapala, Neha Bhavnani, Julie Dougherty, Mahmood Khan, Sailaja Paruchuri, Charles Thodeti

**Affiliations:** ^1^ Department of Integrative Medical Sciences, Northeast Ohio Medical University, Rootstown, OH, United States; ^2^ Department of Physiology and Pharmacology, University of Toledo, Toledo, OH, United States; ^3^ Dorothy M. Davis Heart and Lung Research Institute, The Ohio State University Wexner Medical Center, Columbus, OH, United States; ^4^ Department of Emergency Medicine, The Ohio State University, Columbus, OH, United States

**Keywords:** endothelial cells, extracellular vesicles, transient receptor potential vanilloid 4, tumor angiogenesis, vascular endothelial growth factor receptor 2

## Abstract

Tumor angiogenesis is initiated and maintained by the tumor microenvironment through secretion of autocrine and paracrine factors, including extracellular vesicles (EVs). Although tumor-derived EVs (t-EVs) have been implicated in tumor angiogenesis, growth and metastasis, most studies on t-EVs are focused on proangiogenic miRNAs and growth factors. We have recently demonstrated that conditioned media from human lung tumor cells (A549) downregulate TRPV4 channels and transform normal endothelial cells to a tumor endothelial cell-like phenotype and induce abnormal angiogenesis *in vitro, via* t-EVs. However, the underlying molecular mechanism of t-EVs on endothelial cell phenotypic transition and abnormal angiogenesis *in vivo* remains unknown. Here, we demonstrate that t-EVs downregulate TRPV4 expression post-translationally and induce abnormal angiogenesis by activating Rho/Rho kinase/YAP/VEGFR2 pathways. Further, we demonstrate that t-EVs induce abnormal vessel formation in subcutaneously implanted Matrigel plugs *in vivo* (independent of tumors), which are characterized by increased VEGFR2 expression and reduced pericyte coverage. Taken together, our findings demonstrate that t-EVs induce abnormal angiogenesis *via* TRPV4 downregulation-mediated activation of Rho/Rho kinase/YAP/VEGFR2 pathways and suggest t-EVs and TRPV4 as novel targets for vascular normalization and cancer therapy.

## Introduction

A key step in the formation of solid tumors is the growth of new vessels from pre-existing ones, a process known as tumor angiogenesis. The crosstalk of tumor cells and varying cell types within the tumor microenvironment (TME) is a crucial part of the tumor angiogenic process. However, this crosstalk eventually leads to the abnormal characteristics observed in the tumor vasculature (Lee et al., 2015). Tumor vessels exhibit decreased pericyte coverage and breakdown of basement membranes, leading to hyperpermeability, disorganization, and vessel enlargement (Baluk et al., 2005; Nagy et al., 2006). Importantly, these vascular abnormalities decrease the efficacy of chemotherapies due to poor drug perfusion into the tumors (Saleem and Price, 2008; Dewhirst and Secomb, 2017). Traditional anti-angiogenic therapies target VEGF and show promise as cancer-treating agents, however, due to acquired drug resistance, favorable effects are limited to short-term treatment (Miller et al., 2007; Ratner et al., 2012). Therefore, there is an urgent need for drug targets that aim to “normalize” rather than impede the tumor vasculature.

Crosstalk between cells of the TME is accomplished *via* several autocrine, paracrine, and juxtacrine signaling mechanisms (Ge et al., 2019). Recently, it has become evident that extracellular vesicles (EVs) play key role in both the autocrine and paracrine signaling cascades involved in cancer progression and metastasis (Gangoda et al., 2015; Asare-Werehene et al., 2020). EVs are small nanovesicles (20–5,000 nm) consisting of exosomes, micro-vesicles, and apoptotic bodies, which contain cargo such as nucleic acids, miRNAs, proteins, and lipids (Doyle and Wang, 2019; Veziroglu and Mias, 2020). Once secreted, recipient cells internalize EVs via endocytosis, thus completing the trafficking of cargo (Joshi et al., 2020). In fact, it is well known that the cargo found in/on EVs is involved in the process of tumor angiogenesis. Tumor-derived EVs (t-EVs) have been shown to express heparin-bound VEGF on their surface, which induces endothelial cell migration and tube formation (Ko et al., 2019). Additionally, Skog et al. discovered angiogenic proteins TIMP-2, IL-6, IL-8, TIMP-1, VEGF, and angiogenin in glioblastoma-derived EVs (Skog et al., 2008). Angiogenic promoting microRNA, miR-181b-5p, has been identified in EVs secreted from esophageal squamous cell carcinomas, which can be received by endothelial cells to stimulate angiogenesis *via* PTEN and PHLPP2 targeting (Wang et al., 2020). Despite their known angiogenic effects, the role of t-EVs on endothelial cell transformation is not well understood.

The calcium permeable mechanosensitive ion channel, transient receptor potential vanilloid 4 (TRPV4), has many implications in cancer progression. For instance, it has been shown that knockdown of TRPV4 in breast cancer cells decreases blebbing, a process by which the cellular membrane detaches from the cortex due to pressure, which has implications in metastasis (Lee et al., 2016). Additionally, antagonism of TRPV4 channels in hepatocellular carcinoma (HCC) leads to decreased ERK phosphorylation/activation, a key pathway involved in cell proliferation (Fang et al., 2018). Notably, we have shown that TRPV4 is downregulated in tumor endothelial cells and that pharmacological activation of these channels normalizes the tumor vasculature and improves drug delivery (Adapala et al., 2016). Further, we have shown that the downregulation of TRPV4 increases basal Rho activity, endothelial cell proliferation via ERK phosphorylation, and VEGFR2 and YAP signaling (Thoppil et al., 2015; Thoppil et al., 2016; Kanugula et al., 2019). However, the angiogenic implications of TRPV4 modulation *via* t- EVs remains unknown. To elucidate this, previously, we found that tumor cell conditioned media (TCM) causes normal human endothelial cells (hNEC) to transform into a human tumor endothelial cell-like phenotype (hTEC) *via* TRPV4 downregulation, leading to abnormal tube formation *in vitro*. We also demonstrated that t-EVs isolated from TCM induces the abnormal tube formation seen in hTEC (Guarino et al., 2019). In the present study, we investigated if t-EVs downregulate endothelial TRPV4 channels and modulate various signaling pathways involved in angiogenesis. Further, we examined if t-EVs can induce abnormal angiogenesis *in vivo*, in wild-type (WT) mice, independent of tumors. Our findings suggest that t-EVs modulate the endothelial cell phenotype, TRPV4 channels, and downstream signaling mechanisms, which induces abnormal angiogenesis both *in vitro* and *in vivo*.

## Methods

***Cell culture***: Human microvascular endothelial cells (HMEC-1) were purchased from ATCC (Manassas, VA, United States) and were cultured as previously described (Guarino et al., 2019). Briefly, HMEC-1 were cultured in MCDB-131 media, supplemented with 10% FBS, 1% penicillin-streptomycin, 1% l-glutamine, 1% hydrocortisone, and 10 ng/ml human EGF.

***Extracellular vesicle isolation and characterization***: Adenocarcinomic human alveolar basal epithelial cells (A549) were purchased from ATCC and cultured in normal DMEM high glucose media supplemented with 10% fetal bovine serum (FBS) and 1% penicillin-streptomycin. Once the cells had reached ∼80% confluence, complete media was replaced with serum-free media for 24 h, collected, and spun down to remove any cellular debris. Media was passed through a 0.22 µm syringe filter and stored at −80°C for future use. Extracellular vesicles were isolated and characterized as previously described (Guarino et al., 2019; Dougherty et al., 2020). In brief, TCM was concentrated 5X with 100 kD MWCO centrifugal filters, 1/5 volume of ExoQuick-TC reagent (SBI, Mountain View, CA, United States) was added to TCM, which was then incubated overnight at 4°C, followed by centrifugation at 1,500 x g for 30 min at 4°C. Centrifugation was performed a second time to remove residual supernatant. EVs were re-suspended in sterile PBS and stored at −80°C. For characterization, diluted EVs (PBS) were added to Malvern Nanosight NS300 and analyzed using Nanoparticle Tracking Analysis (NTA) software v3.3 (Malvern, United Kingdom) in triplicate runs. For total EV protein estimation, EVs were lysed in 1x RIPA buffer [150 mM NaCl, 50 mM Tris HCl pH 8.0, 5 mM EDTA, 10% v/v IGEPAL CA-630, 5% w/v sodium deoxycholate, 1% w/v sodium dodecyl sulfate, 1x comlplete protease inhibitor cocktail (Roche)] on ice for at least 10 min. EVs were appropriately diluted with PBS and protein estimation utilized the Protein Assay Dye Reagent Concentrate (BioRad), with absorbance measured on a spectrophotometer at 595nm, and interpolation to a BSA standard curve. EVs were tested in technical triplicate.

***Calcium imaging***: HMEC-1 cells were cultured as described above on MatTek glass bottom dishes (MatTek, Ashland, MA, United States). After 24 h, complete media was replaced with a combination of complete media and serum free media (25:75) and t-EVs were added to cells at a concentration of 100 μg/ml. After 48 h, cells were loaded with Fluo-4/AM (4 µM) for 25 min and were washed in previously described calcium media (Adapala et al., 2011; Adapala et al., 2016). Live cell imaging was done on an Olympus FluoView 300 microscope (Olympus, Shinjuku, Tokyo, Japan) after stimulation with GSK1016790A (100 nM), the TRPV4 agonist.

***2D angiogenesis assays (in vitro)***: Growth factor reduced Matrigel^®^ (BD biosciences, San Jose, CA, United States) was plated on a 48-well plate and placed at 37°C for 30 min (Adapala et al., 2016; Thoppil et al., 2016; Guarino et al., 2019). HMEC-1 were cultured and treated with 100 μg/ml of t-EVs for 48 h prior to angiogenesis assays, as described above. The Rho kinase inhibitor, Y27632, was added to cells (10 µM) just before plating on Matrigel. Images were taken at 24 h after plating the cells on Matrigel and tube length was quantified using ImageJ Software.

***Immunocytochemistry***: Cells were cultured in 6-well plates on glass coverslips and fixed with 4% paraformaldehyde (PFA) for 20 min. Cells were then washed 3x in—PBS, permeabilized for 15 min with 0.25% TritonX-100, blocked for 30 min in FBS-containing media, and incubated with VEGFR2 primary antibody (1:200; Cell Signaling Technology, Danvers, MA, United States) or YAP primary antibody (1:100; Santa Cruz Biotechnology, Dallas, TX, United States) for 1 h at room temperature. Following incubation, cells were again washed 3x in PBS, followed by incubation with appropriate Alexa Fluor (488 and 594) conjugated antibody (1:500; Thermo Fisher Scientific, Waltham, MA, United States). Cells were washed 3x in PBS and mounted with DAPI containing mounting media (Vector Laboratories, Burlingame, CA, United States) on glass slides. Images were obtaining using TCS SP5 laser scanning confocal microscope with MP at 63x (Leica Microsystems, Germany) and processed using ImageJ (NIH, Bethesda, Maryland, United States) software.

***Western Blot***: Cells were lysed in RIPA buffer containing 1X protease and phosphatase inhibitor cocktail (Millipore Sigma and Roche, Basel, Switzerland). Samples were prepared with 4X Laemmli sample buffer (Bio-Rad, Hercules, CA, United States) supplemented with *β*-mercaptoethanol. Lysates were loaded into 7.5% Mini-PROTEAN^®^ TGX™ precast polyacrylamide gels (Bio-Rad, Hercules, CA, United States) for electrophoresis. Gels were transferred onto a PVDF membrane, which was briefly activated in methanol, and was blocked with 5% milk powder in tris-buffered saline with 0.1% Tween-20 (TBST). Membranes were incubated in primary antibodies (TRPV4, 1:300) (Alamone Labs, Jeru-salem, Israel; pVEGFR2 (1:1,000), VEGFR2 (1:1,000), and GAPDH, (1:1,000) Cell Signaling Technology, Danvers, MA, United States) overnight at 4°C. The next day, membranes were washed in 1X TBST 3x for 15 min and incubated with secondary antibody, goat anti rabbit (1:5,000) conjugated with horseradish peroxidase (Cell Signaling Technology, Danvers, MA, United States). Signals were detected with Luminata Crescendo (EMD Millipore, Burlington, MA, United States) and developed with a FluorChem M Simple Imager (Protein Simple, San Jose, CA, United States). Quantification of proteins was performed using ImageJ software (NIH, Bethesda, Maryland, United States).

***In vivo Matrigel plug assays***: All experiments were performed according to the approved protocol by the Institutional Animal Care and Use Committee (IACUC) at Northeast Ohio Medical University. t-EVs were isolated and characterized as described above. Phenol red free Matrigel was mixed with 0.25 μg/ml bFGF, 0.2 ng/ml mouse VEGF, and 0.58 μl/ml heparin (diluted in saline). 50µg/plug of EVs were added to Matrigel mixture and were mixed with an ice-cold pipette. C57BL/6 mice were anesthetized with isoflurane and were injected subcutaneously with 500 µl of the pre-mixed Matrigel solution in each flank region (2 injections per mouse). After 14 days post-injection, mice were euthanized with Fatal-Plus and plugs were harvested for histological analysis.

***Immunohistochemistry*** *:* Matrigel plugs were harvested from mice 14 days post-injection and were immediately frozen in OCT for cryo-sectioning. Samples were sectioned (10 µm) using a cryo-stat and were permeabilized in ice-cold acetone. Next, slides were washed in 1X TBS 3x for 5 min each and then blocked with 5% normal donkey serum or 5% goat serum. After blocking the following primary antibodies were added: CD31 (1:50; Invitrogen, Waltham, MA, United States), NG2 (1:100; EMD Millipore, Burlington, MA, United States), VEGFR2 (1:200; Cell Signaling Technologies, Danvers, MA, United States). Slides were incubated in a humidified chamber overnight at 4°C, washed 3x in 1X TBS, and incubated with appropriate AlexaFluor (488 and 594) secondary antibodies (1:500). After washing 3x in 1X TBST, slides were mounted with DAPI containing mounting medium (Vector Laboratories, Burlingame CA, United States). Images were acquired using an IX81 Olympus microscope with a Fluoview FV1000 or confocal laser scanning system and fluorescence intensities were quantified using ImageJ (NIH, Bethesda, Maryland, United States) software.

***RT-Quantitative PCR (qPCR)***: RNA was isolated from endothelial cells with the RNeasy Mini Kit (Qiagen, Hilden, Germany) and was quantified using Take3™ Micro-Volume Plate on the Epoch™ Microplate Spectrophotometer (BioTek Instruments, Inc., Winooski, VT, United States). cDNA synthesis was performed using the RevertAid First Strand cDNA Synthesis Kit (Thermo Fisher Scientific, Waltham, MA, United States). qPCR was done on the Fast-Real-Time PCR system using SYBR green master mix (Thermo Fisher Scientific, Waltham, MA, United States). The following real-time primer sets were purchased from Integrated DNA Technologies (Coralville, IA, United States): *β*-actin (forward- 5′-ACG​TTG​CTA​TCC​AGG​CTG​TG-3′, reverse-5′-GAGGGCATACCCCTCGTAGA-3′) TRPV4 (forward- 5′-TCA​CTC​TCA​CCG​CCT​ACT​ACC​A-3’: reverse- 5′-CCC​AGT​GAA​GAG​CGT​AAT​GAC​C-3′). mRNA expression was normalized to housekeeping gene, *β*-actin, and ΔΔCt values were expressed as a fold change relative to untreated ECs.

***Statistical analysis***: All data was analyzed with independent sample *t*-test using SPSS V. 24 software. The significance was set at *p ≤ 0.05; **p ≤ 0.01; ***p ≤ 0.001; ****p ≤ 0.0001. All values were expressed as means ± SEM.

## Results

### Tumor-Derived EVs (t-EVs) Downregulate Endothelial TRPV4 Channels Post-translationally

**FIGURE 1:**
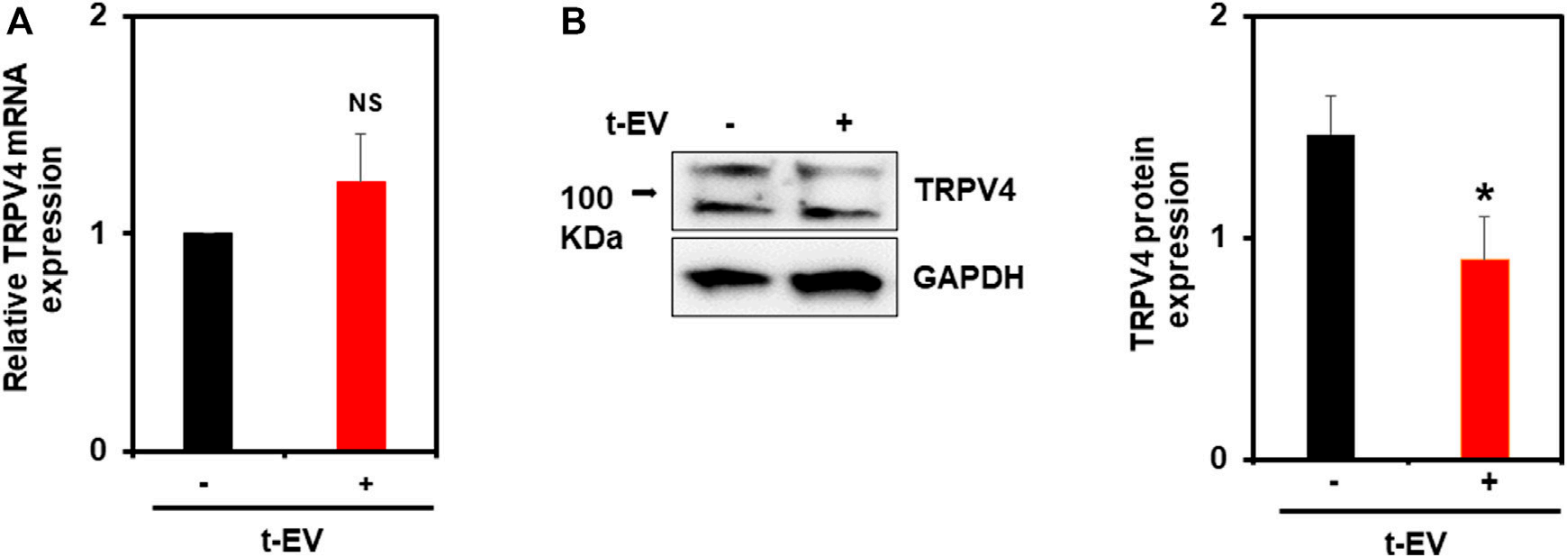
t-EVs cause post-translational downregulation of endothelial TRPV4 channels. **(A)** qPCR analysis of TRPV4 mRNA expression from t-EV treated (+) or untreated (−) ECs. Note, no significant difference of TRPV4 mRNA between treated and untreated ECs (NS). TRPV4 mRNA expression was normalized to housekeeping gene, *β*-actin. **(B)** Representative western blots depicting decreased TRPV4 protein expression in t-EV treated ECs compared to untreated cells. Quantitative analysis demonstrating significantly decreased TRPV4 protein expression in t-EV treated ECs (*p ≤ 0.05; *n* = 7). Protein expression was normalized to GAPDH. The results shown are a mean ± SEM from three independent experiments.

We previously showed that tumor cell conditioned media (TCM) causes abnormal tube formation *in vitro* (via downregulation of TRPV4), and that the exosome inhibitor, GW4869, attenuates this effect (Guarino et al., 2019). Here, we assessed the expression of endothelial TRPV4 channels after exposure to purified t-EVs (Figures 1A,B). We found no significant differences in TRPV4 mRNA expression between t-EV-treated and control ECs (Figure 1A). However, western blot analysis revealed significantly decreased TRPV4 protein expression in t-EV-treated ECs compared to control ECs (Figure 1B, *p* ≤ 0.05). These results indicate that t-EVs induce TRPV4 downregulation post-translationally.

### t-EVs Modulate Rho/Rho Kinase/YAP/VEGFR2 Signaling *via* Downregulation of TRPV4 Channels

**FIGURE 2:**
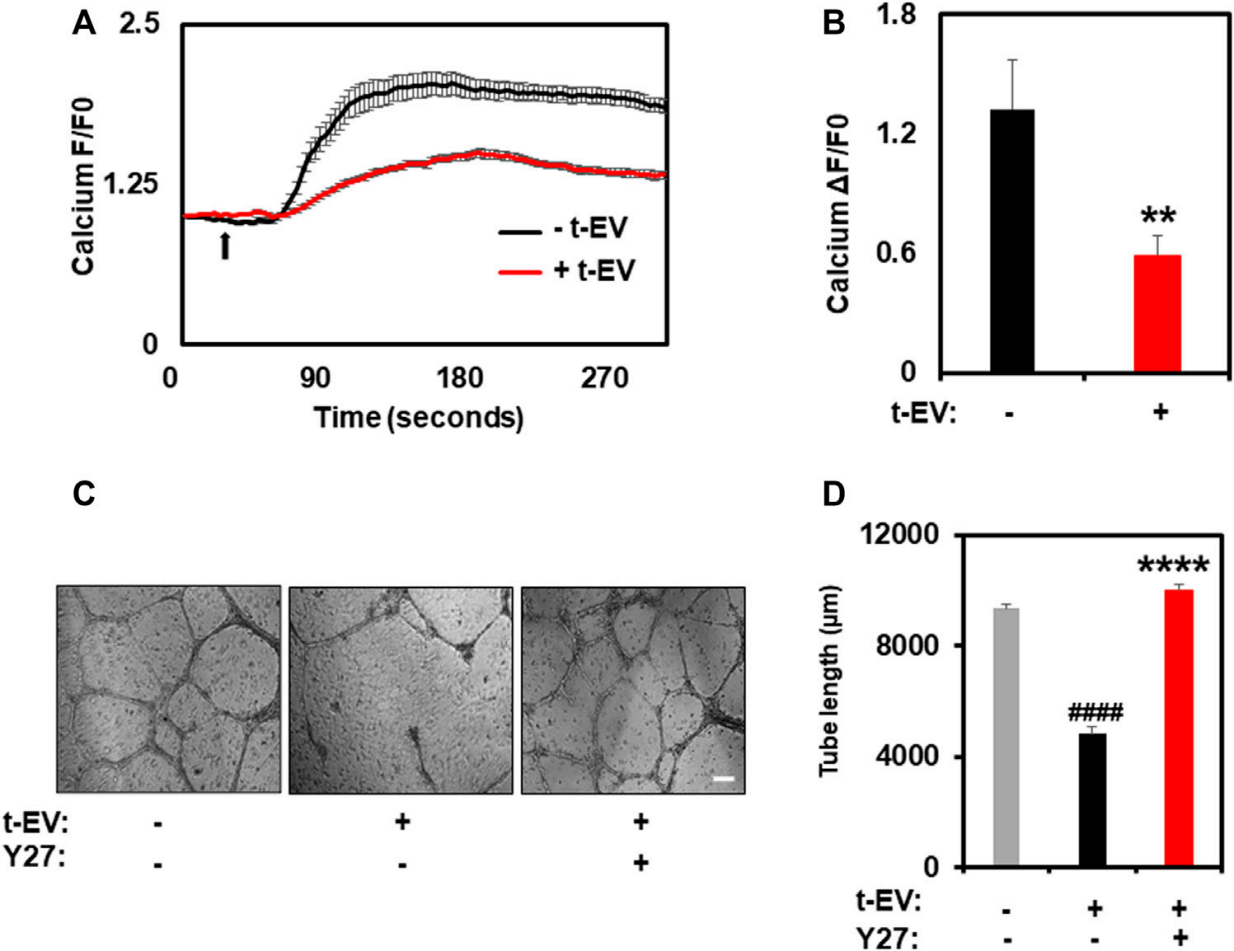
t-EV treatment decreases functional activity of TRPV4 channels and inhibition of Rho kinase normalized t-EV induced abnormal angiogenesis. **(A)** Average traces showing calcium influx in response to the TRPV4 agonist, GSK1016790A (100 nM), in Fluo-4 loaded ECs treated with (+t-EV) or without (−t-EV) t-EVs. The arrow denotes time of stimulation with TRPV4 agonist. **(B)** Quantitative analysis of calcium influx showing a significant reduction (****p ≤ 0.01) in TRPV4-mediated calcium influx in + t-EV ECs compared to− t-EV ECs. (F/F0 = ratio of normalized fluorescent intensity relative to time 0). **(C)** Phase contrast micrographs (4x) of tube formation by ECs exposed to t-EVs with or without the Rho kinase inhibitor, Y-27632 (Y27) plated on 2D Matrigels. Scale bar = 200 μm. **(D)** Quantitative analysis of tube length demonstrating significantly increased tube length (****p ≤ 0.0001 indicates statistical significance relative to control cells; ####*p* ≤ 0.0001 indicates statistical significance relative to t-EV-treated ECs without Y27; no statistical significance was observed between control ECs and t-EV/Y27 ECs) in + t-EV cells treated with the Rho kinase inhibitor, Y27, indicating vascular normalization. The results shown are a mean ± SEM from three independent experiments.

**FIGURE 3:**
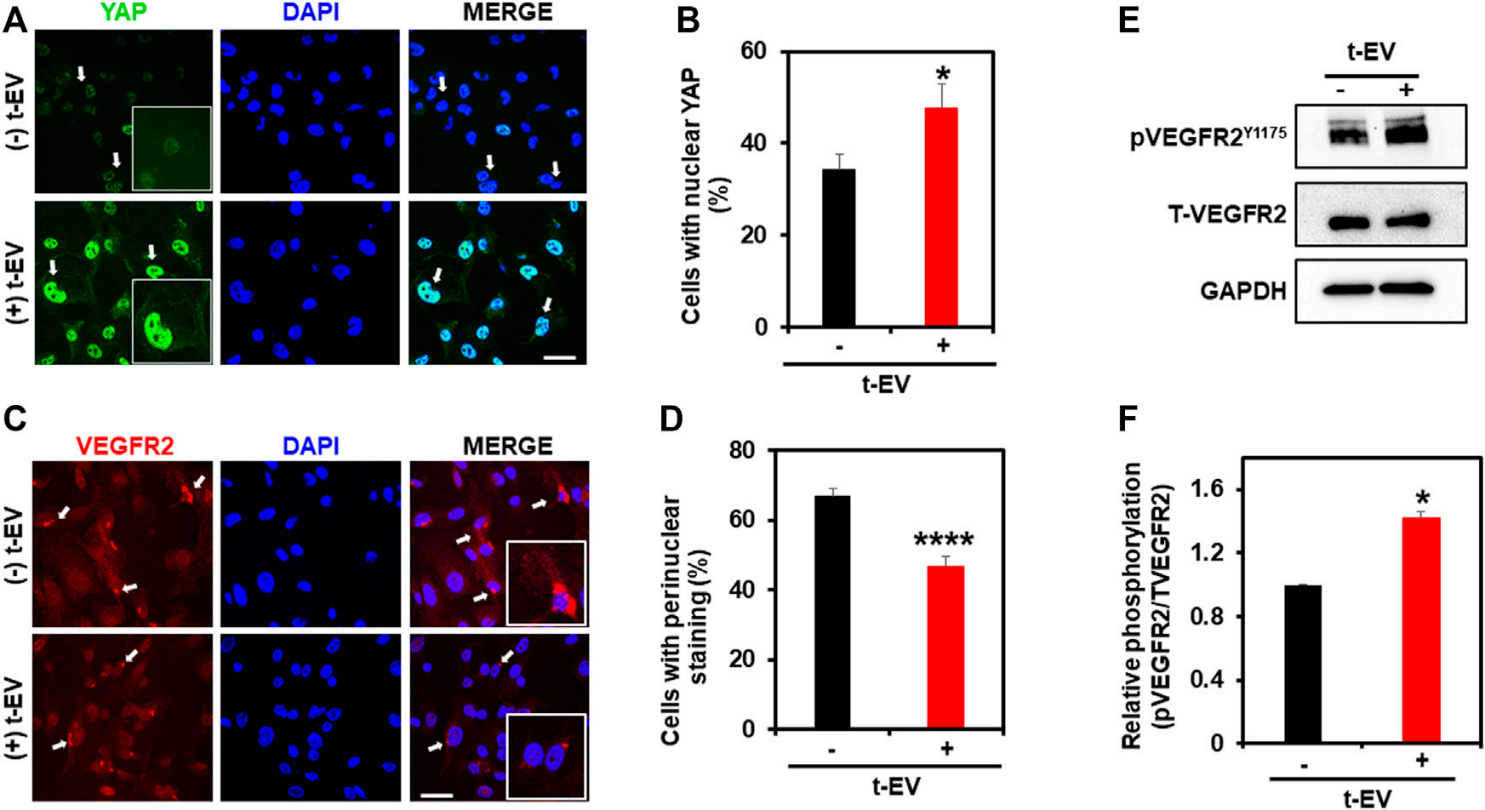
t-EV treatment activates YAP nuclear translocation and reduces perinuclear VEGFR2 in ECs. **(A)** Representative confocal immunofluorescence images (63x) showing YAP (green) localization in t-EV-treated and untreated ECs. Cell nuclei were stained with DAPI (blue). Scale bar = 25 μm. Note, strong nuclear YAP localization (arrows) in t-EV-treated ECs (arrows; inset: zoomed image). **(B)** Quantitative analysis of the percent of cells with YAP nuclear staining, with or without t-EV treatment (*n* = 180–240 cells/condition). Note, the significantly increased (*p ≤ 0.05) percent of ECs with YAP nuclear localization in + t-EV cells compared to − t-EVs. The results shown are a mean ± SEM from three independent experiments. **(C)** Representative confocal immunofluorescence images (63x) showing VEGFR2 (red) localization in t-EV treated and untreated ECs. Cell nuclei were stained with DAPI (blue). Scale bar = 25 μm. Note, strong perinuclear VEGFR2 localization (arrows; inset: zoomed image) in untreated ECs, which is diminished after t-EV exposure. **(D)** Quantitative analysis of the percent of cells with perinuclear VEGFR2 in the presence or absence of t-EVs. Note, the significantly decreased (****p ≤ 0.0001) percent of ECs with perinuclear VEGFR2 in + t-EV compared to − t-EVs. The results shown are a mean ± SEM from three independent experiments. **(E)** Representative immunoblots showing the phosphorylation of VEGFR2 at Y1175 in the presence or absence of t-EVs in human microvascular endothelial cells **(Top panel)**. Total VEGFR2 and GAPDH are served as controls. **(F)** Quantitative analysis of immunoblots showing T-EV treatment enhanced the VEGFR2 phosphorylation compared to controls. The results shown are a mean ± SEM from two independent experiments.

To unequivocally confirm that t-EVs downregulate functional expression of TRPV4, we next performed calcium imaging to assess the activity of TRPV4 in ECs. Indeed, calcium imaging revealed significantly decreased calcium influx in ECs exposed to t-EVs in the presence of the TRPV4 agonist, GSK1 (Figures 2A,B; *p* ≤ 0.01), compared to untreated ECs. We have previously shown that the downregulation of TRPV4 causes abnormal tube formation via increased Rho/Rho kinase activity (Thoppil et al., 2016). Therefore, we asked whether t-EV-dependent downregulation of TRPV4 modulates angiogenesis via Rho/Rho kinase signaling. Here, we confirm that t-EVs cause abnormal tube formation in ECs (Figures 2C,D; middle panel) as evidenced by collapse of tubular net-work compared to control EC. However, when t-EV-exposed ECs are treated with the Rho kinase inhibitor, Y27632, tube formation is normalized (Figures 2C,D, right panel; *p* ≤ 0.0001). Next, we investigated the effects of t-EVs on YAP and VEGFR2 signaling. Yes-associated protein (YAP) is an important transcriptional coactivator that has been shown to localize to the nucleus of ECs during angiogenesis (He et al., 2018) and is known to be regulated by Rho/Rho kinase (Dupont et al., 2011; Yu et al., 2012; Ohgushi et al., 2015). Therefore, we asked if exposure to t-EVs mediates YAP nuclear localization. Immunostaining revealed significantly increased YAP nuclear localization in t-EV-treated ECs compared to untreated ECs (Figures 3A,B; *p* ≤ 0.05; arrows and inset). Next, we found that VEGFR2 is mostly localized to perinuclear regions in un-treated ECs, which showed significant reduction in t-EV treated cells (Figures 3C,D; *p* ≤ 0.0001; arrows and inset), consistent with our previous findings (Guarino et al., 2019; Kanugula et al., 2019). To independently confirm VEGFR2 activation, we have measured VEGFR2 phosphorylation at Y1175 in ECs treated or untreated with t-EVs. We found that t-EVs increased VEGFR2 phosphorylation suggesting the activation of VEGFR2 (Figures 3E,F; *p* ≤ 0.05). Taken together, these data indicate that t-EVs downregulate the functional activity of TRPV4, and subsequently modulate Rho/Rho kinase/YAP/VEGFR2 pathways.

### t-EVs Induce Abnormal Angiogenesis Independent of Solid Tumors

**FIGURE 4:**
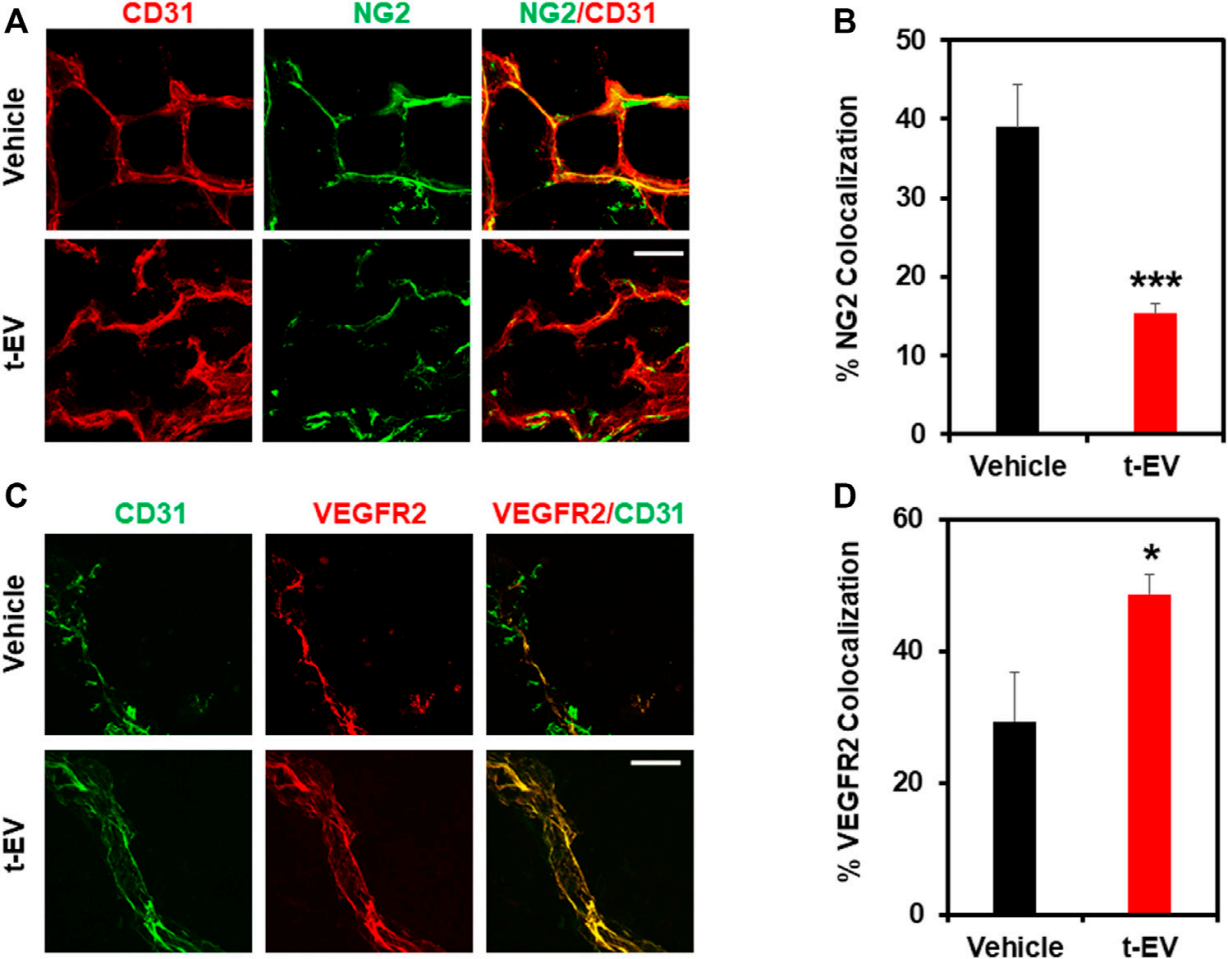
t-EVs cause abnormal angiogenesis *in vivo* as evidenced by decreased pericyte coverage and increased VEGFR2 expression. **(A)** Representative immunofluorescence images (60x) of Matrigel sections stained with endothelial marker CD31 (red) and pericyte marker NG2 (green). Note, the reduced pericyte coverage and disorganized appearance of t-EV treated vessels. Scale bar = 100 μm. **(B)** Quantitative analysis revealing significantly decreased NG2/CD31 colocalization in t-EV Matrigel plugs (*n* = 7) (***p ≤ 0.001) compared with vehicle alone (*n* = 4). **(C)** Representative immunofluorescence images (60X) taken from Matrigel plugs stained with endothelial marker CD31 (green) and VEGFR2 (red). Scale bar = 100 μm. **(D)** Quantitative analysis demonstrating significantly increased VEGFR2/CD31 colocalization (*p ≤ 0.05) in t-EV treated Matrigel plugs (*n* = 7) compared to the vehicle (*n* = 4).

Based on our *in vitro* results, we investigated the effects of t-EVs on vessel growth *in vivo*, independent of tumors. To accomplish this, we injected Matrigel plugs with or without t-EVs (vehicle) into 8-week-old C57BL/6 WT mice. After 14 days, Matrigel plugs were removed and analyzed for vessel structure/integrity via immunohistochemistry for EC marker CD31, VEGFR2, and pericyte marker NG2 (Figures 4A–D; *p* ≤ 0.05). We found vessels with distinct colocalization of NG2/CD31 in vehicle-treated Matrigel plugs, however, NG2/CD31 colocalization was reduced significantly in t-EV treated Matrigel plugs (Figures 4A,B; *p* ≤ 0.001), indicative of decreased pericyte coverage. Next, we found significantly increased VEGFR2/CD31 colocalization in t-EV treated plugs compared to the vehicle (Figures 4C,D; *p* ≤ 0.05), indicating that t-EVs compromise vessel integrity by causing increased expression of VEGFR2 and decreased pericyte coverage in otherwise healthy vessels.

## Discussion

In the current study, we demonstrate that tumor derived-EVs downregulate endothelial TRPV4 channels post-translationally and induce abnormal angiogenesis via Rho/Rho kinase/YAP/VEGFR2 pathway. Further, we demonstrate that independent of tumors, t-EVs stimulate a tumor angiogenesis-like phenotype in Matrigel plugs *in vivo*, which is evidenced by increased VEGFR2 positive vessels with reduced pericyte coverage.

The vasculature of solid tumors is hyperpermeable and tortuous, which makes drug delivery difficult (Ma and Waxman, 2008; Stylianopoulos and Jain, 2013). Although vascular normalization improves the abnormal characteristics of tumor vessels and makes drug delivery more efficient (Mpekris et al., 2020; Moradi Kashkooli et al., 2021), these modalities are still dependent on classical anti-VEGF therapies. We have previously shown that the expression of mechanosensitive ion channel, TRPV4, is downregulated in tumor endothelial cells and activation of TRPV4 normalizes the tumor vasculature and improves chemotherapy (Adapala et al., 2016). However, the mechanisms by which tumors or the tumor microenvironment downregulate TRPV4 channels and influences angiogenesis *in vivo*, are not known. Here, we investigated the effects of t-EVs on endothelial TRPV4 channels and modulation of downstream signaling pathways, as well as angiogenesis *in vitro* and *in vivo*.

We have previously demonstrated that conditioned media (TCM) from human lung cancer cells transformed normal human endothelial cells into a tumor endothelial cell-like (TEC) phenotype via downregulation of TRPV4 channels (Guarino et al., 2019). Further, pre-treatment with exosome inhibitor GW4869 inhibited the TEC-like phenotype induced by TCM, indicating the role of t-EVs in this process. Indeed, treatment with purified t-EVs induced abnormal tube formation *in vitro*. Consistent with our previous findings that TCM downregulates TRPV4 expression and activity, purified t-EVs induced TRPV4 downregulation in ECs. Importantly, TRPV4 expression is reduced at the protein level but not at the mRNA level suggesting that t-EVs modulate TRPV4 expression, post-translationally. Calcium imaging experiments further confirmed the functional downregulation of TRPV4.

We have previously shown that downregulation or deletion of TRPV4 increases basal Rho/Rho kinase activity and induces abnormal angiogenesis (Thoppil et al., 2016). Indeed, we found that treatment with t-EVs induced abnormal tube formation, which was normalized by Rho kinase inhibitor, Y27632. YAP, a key transcriptional co-activator of the Hippo signaling pathway, is known to translocate to the nucleus in endothelial cells during angiogenesis (Hooglugt et al., 2021). Further, YAP was shown be activated by actin polymerization induced by Rho/Rho kinase (Dupont et al., 2011; Yu et al., 2012; Ohgushi et al., 2015). Importantly, VEGF has also been shown to stimulate nuclear translocation of YAP/TAZ and YAP/TAZ regulates VEGFR2 cellular localization/trafficking (Wang et al., 2017). The present study revealed significantly increased nuclear localization of YAP in ECs that were exposed to t-EVs. Moreover, we observed a reduction in perinuclear VEGFR2 after exposure to t-EVs, which is indicative of its plasma translocation and activation (Manickam et al., 2011). To delineate the molecular mechanism by which t-EVs downregulate endothelial TRPV4 channels, we checked TRPV4 expression at both the mRNA and protein level. Interestingly, there was no significant difference in TRPV4 mRNA expression, however, TRPV4 protein expression was significantly downregulated. Since TRPV4 channels have been shown to undergo internalization via ubiquitination (Wegierski et al., 2006; Shukla et al., 2010), it is possible that t-EVs contain cargo that tag TRPV4 for ubiquitination, however, further studies are needed to determine the exact mechanisms by which t-EVs downregulate TRPV4 protein expression.

**FIGURE 5:**
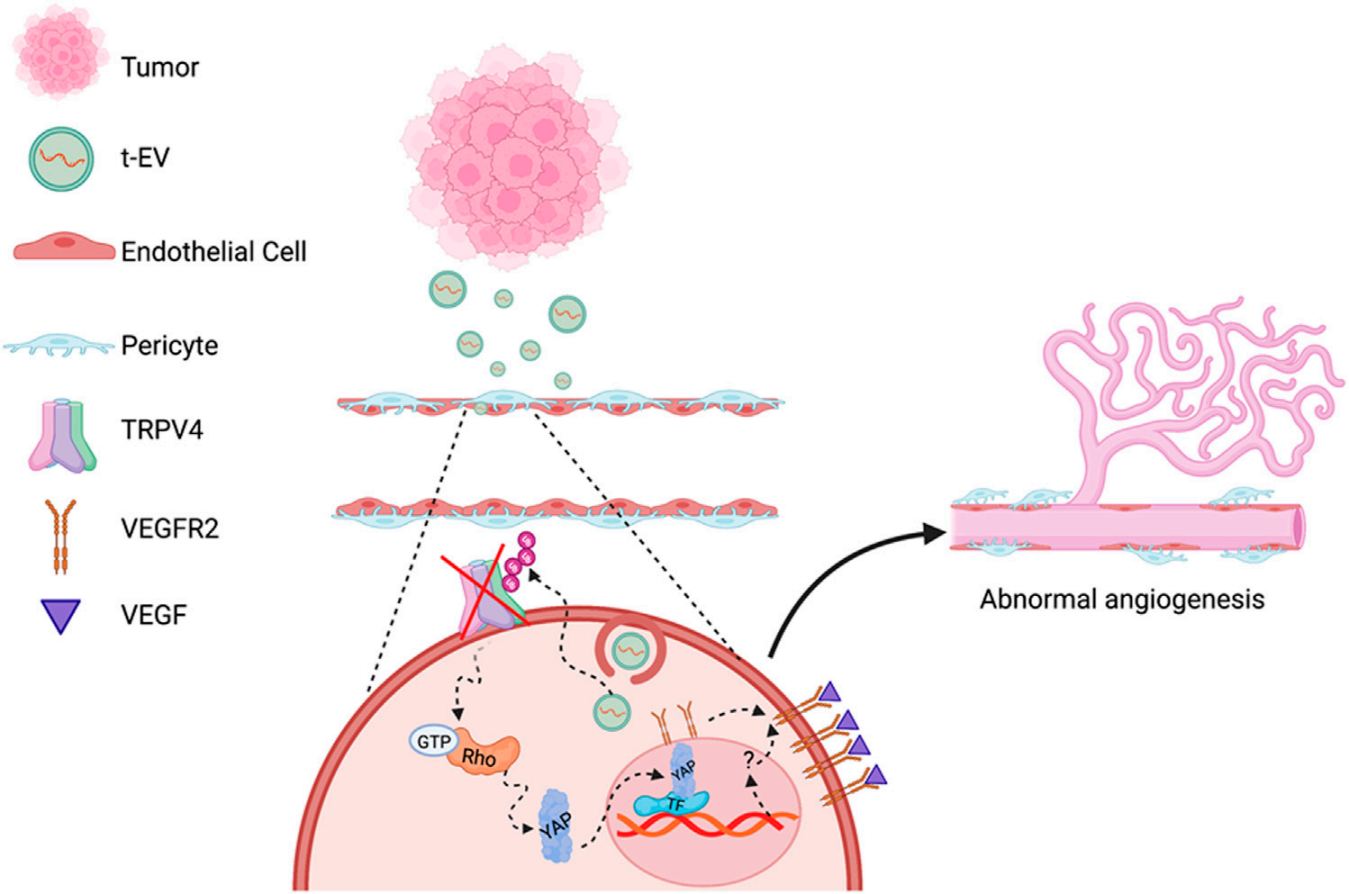
Schematic of proposed molecular mechanisms of TRPV4 downregulation mediated abnormal angiogenesis. EVs secreted by tumors (t-EVs) released into the tumor microenvironment act on endothelial cells, leading to the downregulation of TRPV4 channels may be via ubiquitination. TRPV4 down-regulation, subsequently, induces Rho/Rho kinase mediated YAP nuclear translocation leading to VEGFR2 translocation from the perinuclear region to the plasma membrane (Kanugula et al., 2019). YAP commonly binds to transcription factors (TF) that initiate gene expression involved in cellular growth and proliferation (Huh et al., 2019), however, the mechanism by which YAP activates VEGFR2 translocation is unknown. VEGFR2 activation by VEGF, further induces EC proliferation, migration, and eventually, abnormal angiogenesis (Figure created with Biorender.com).

In the current study, we translated our *in vitro* findings to *in vivo* models of angiogenesis. When Matrigel plugs combined with t-EVs were implanted into WT mice, we found decreased pericyte coverage and increased VEGFR2 colocalization with the endothelium, which is reminiscent of pathological angiogenesis (Bergers and Song, 2005). The data presented in this study show, for the first time, that t-EVs downregulate the functional expression of TRPV4 and promote dysfunctional vascular growth independent of solid tumor formation. Moreover, we show that this is accomplished via Rho/Rho kinase/YAP/VEGFR2 signaling mechanisms preceding abnormal angiogenesis (Figure 5). Pathological angiogenesis is a hallmark of many diseases, including cancers, retinopathies, and rheumatoid arthritis (Aldebasi et al., 2013; Fallah et al., 2019). Traditional anti-angiogenic drugs targeting VEGF signaling pathways are often used to combat the abnormal angiogenesis observed in disease states by employing various mechanisms of action, including monoclonal anti-bodies, inhibition of tyrosine phosphorylation, decoy VEGF receptors, or ribosomal targeting (Cardones and Banez, 2006). However, these therapies have shown limited success, particularly in cancer (Miller et al., 2007; Ratner et al., 2012). Therefore, it is becoming increasingly important to explore other drug targets of tumor angiogenesis. Thus, our results indicate that both TRPV4 and t-EVs could prove to be novel targets for cancer therapies.

## Data Availability

The raw data supporting the conclusion of this article will be made available by the authors, without undue reservation.
